# Isolated optic neuritis with positive glial fibrillary acidic protein antibody

**DOI:** 10.1186/s12886-023-02927-z

**Published:** 2023-04-26

**Authors:** Nan Jia, Jiawei Wang, Yuhong He, Zhong Li, Chuntao Lai

**Affiliations:** Department of Neurology, Beijing Tongren Hospital, Capital Medical University, Beijing, China

**Keywords:** Optic neuritis, Autoimmune glial fibrillary acidic protein astrocytopathy, Antibody, Behcet’s syndrome

## Abstract

**Background and objectives:**

Autoimmune glial fibrillary acidic protein (GFAP) astrocytopathy (GFAP-A) has been reported as a spectrum of autoimmune, inflammatory central nervous system disorders. Linear perivascular radial gadolinium enhancement patterns on brain magnetic resonance imaging (MRI) are a hallmark of these disorders. GFAP-A is associated with cerebrospinal fluid (CSF) GFAP antibody (GFAP-Ab), while the association with serum GFAP-Ab is less clear. This study aimed to observe the clinical characteristic and MRI changes of GFAP-Ab-positive optic neuritis (ON).

**Methods:**

We performed a retrospective, observational case study at the department of neurology, Beijing Tongren Hospital, from December 2020 to December 2021. The serum of 43 patients and CSF samples of 38 patients with ON were tested for GFAP-Ab by cell-based indirect immune-fluorescence test.

**Results:**

Four patients (9.3%) were detected GFAP-Ab positive, and in three out of the four patients, GFAP-Abs were detected only in serum. All of them demonstrated unilateral optic neuritis. Three patients (1, 2, and 4) experienced severe visual loss (best corrected visual acuity ≤ 0.1). Two patients (2 and 4) had experienced more than one episode of ON at the time of sampling. MRI showed optic nerve hyperintensity on T2 FLAIR images in all GFAP-Ab positive patients, and orbital section involvement was the most common. During follow-up (mean 4.5 ± 1 months), only Patient 1 had a recurrent ON, and no patient developed new other neurological events or systemic symptoms.

**Conclusion:**

GFAP-Ab is rare in patients with ON and may manifest as isolated, relapsing ON. This supports the notion that the GFAP-A spectrum should comprise isolated ON.

## Background

In recent years, autoimmune glial fibrillary acidic protein (GFAP) astrocytopathy (GFAP-A) has been considered an autoimmune inflammatory disease that damages the central nervous system [1]. Perivascular radial enhancement is characteristic of this disease on brain magnetic resonance imaging (MRI) [2]. Viral infection, tumors, and autoimmune disease have been proposed as possible pathogenic mechanisms of GFAP-A. GFAP-A is associated with cerebrospinal fluid (CSF) GFAP antibody (GFAP-Ab), while the association with serum GFAP-Ab is less clear. The clinical manifestation of GFAP-A is quite extensive. Fever, headache, encephalopathy, and other clinical syndromes of meningoencephalitis are common in patients with this disease [2–4]. Some GFAP-A patients also present with dyskinesia, psychiatric disorder, epileptic seizure, autonomic nerve dysfunction [2, 3], area postrema syndrome [5], and isolated myelitis [4]. However, visual loss is rarely reported as the predominant symptom despite bilateral disc edema [6]. In the current study, we report the frequency of GFAP-Ab-positive in patients with optic neuritis (ON) and defined the clinical and MRI changes of GFAP-Ab-positive ON.

## Methods

### Design and study population

A retrospective, observational study was conducted on 43 consecutive patients diagnosed with ON admitted to the Department of Neurology of Beijing Tongren Hospital, Capital Medical University, from December 2020 to December 2021. Inclusion criteria comprised meeting the clinical diagnostic criteria for ON [7, 8] and performing serum and/or CSF GFAP-Ab tests by cell-based indirect immune-fluorescence assay. Those with serum and/or CSF aquaporin-4 (AQP4)-antibody (Ab) or myelin oligodendrocyte glycoprotein (MOG)-Ab positive, as well as those with multiple sclerosis (MS)-ON, have been excluded from the study. Non-arteritic ischemic optic neuropathy, compressive optic neuropathy, hereditary optic neuropathy, radiation optic neuropathy, and infectious ON were also excluded. Of the 43 enrolled patients, 38 (88.4%) tested both serum and CSF for GFAP-Ab, and 5 (11.6%) tested only serum (CSF unavailable). The time from the last onset to sampling was 37.6 ± 25.2 days, and 2 patients were sampled during the acute phase (< 7 days from symptom onset). 22 patients were sampled after corticosteroid treatment. Each patient underwent an examination by both an ophthalmologist and a neurologist. The medical records of patients were reviewed for the characteristics of best corrected visual acuity (BCVA), visual field (VF), optical coherence tomography (OCT), fundus fluorescein angiography (FFA), MRI findings, serology, and CSF parameters. Tumor screening included lung computerized tomography (CT) and abdominal ultrasound was performed in all patients. One GFAP-Ab-positive patient performed positron emission tomography-computed tomography (PET-CT) because of pulmonary nodules. Serum GFAP-Ab titers, MRI, and ophthalmic examinations of patients with positive GFAP-Ab were followed up for a short term.

### MRI acquisition and analysis

Optic nerve and brain MRI (3.0T, Ingenia, Philips Healthcare, the Netherlands) were undergone by all patients. T2/fluid-attenuated inversion recovery (FLAIR), T1 weighted turbo spin echo (TSE-T1) (before and after Gadolinium [Gd] administration), and diffusion-weighted sequences were analyzed to observe the lesion location and characteristics.

### Serology and CSF analysis

The laboratory testing included complete blood cell count, erythrocyte sedimentation rate, C-reactive protein, rheumatoid factor, antinuclear Ab (ANA), anti-double-stranded DNA (dsDNA) Ab, extractable nuclear Ab, angiotensin-converting enzyme, anti-neutrophil cytoplasmic Ab, anticardiolipin Ab, human leukocyte antigen B27, human leukocyte antigen B51, cytokines, thyroid function and Ab, malignant tumor markers (alpha-fetoprotein, AFP; carcinoembryonic antigen, CEA; cancer antigen (CA)-125; CA-199; CA-153, treponema pallidum Ab, human immunodeficiency virus Ab, and paraneoplastic Ab (anti-Hu, Yo, Ri, CV2, Ma2/Ta, amphiphysin). CSF testing included the lumbar puncture opening pressure, cell count, glucose and protein levels, intrathecal immunoglobin G (IgG) synthesis rate, oligoclonal band, and presence of bacteria, viruses, mycobacterium tuberculosis, and cryptococcus.

### GFAP-Ab analysis

**Fig. 1:**
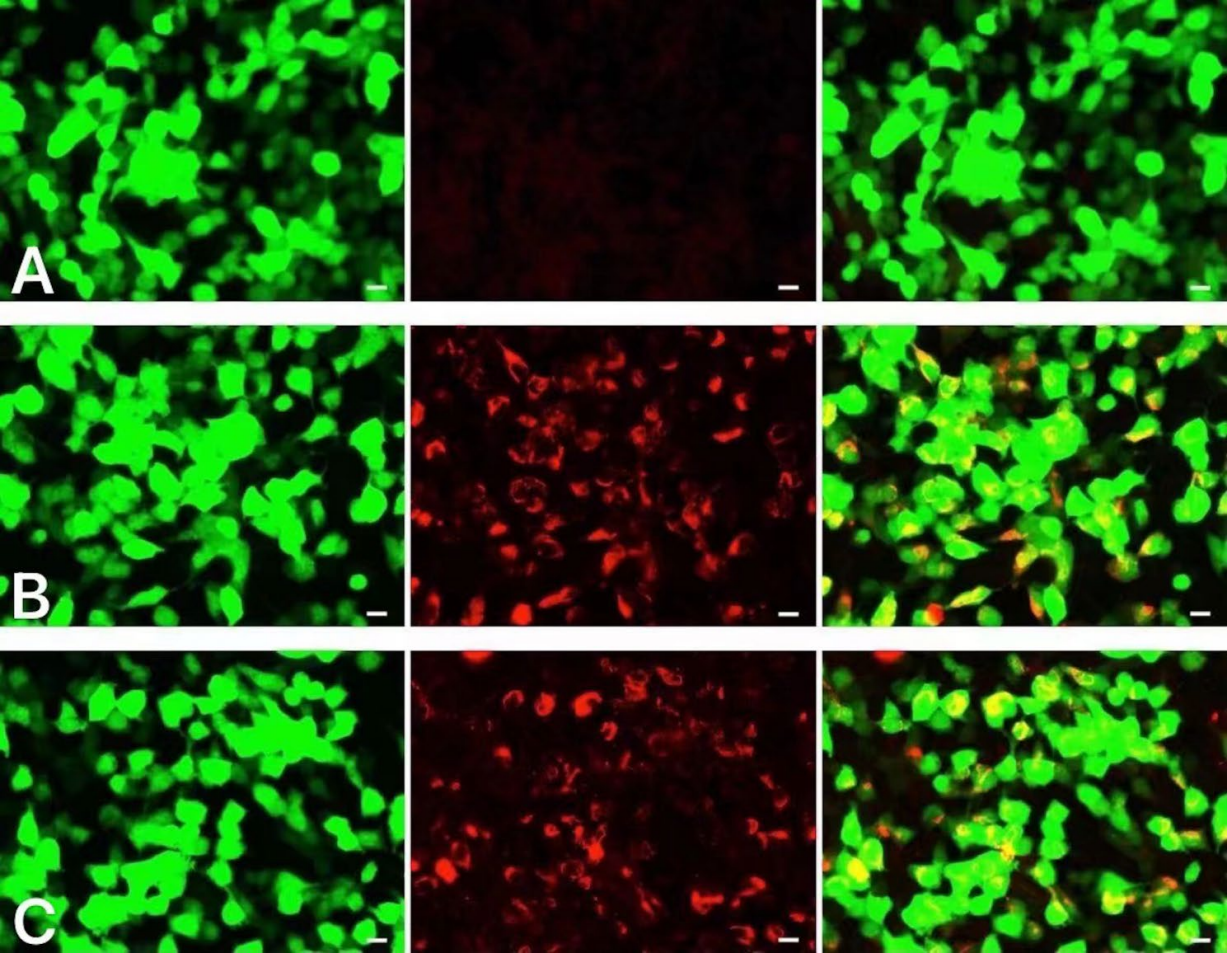
GFAP antibody testing in serum and CSF. GFAPα-IgG in serum/CSF was tested using a cell-based indirect immune-fluorescence test. HEK293 cells were co-transfected with human GFAPα and pcDNA3.1-EGFP (scale bar: 20 μm). Binding of IgG from patient’s serum/CSF using an anti-human IgG antibody (red) to cells transfected with GFAPα (green); merged images (yellow). (**A**): Negative control. (**B**): Positive control. (**C**): Sample from patient 4: GFAPα-IgG was positive in serum/CSF (titer at 1:32).

GFAP-Ab was tested using a fixed cell-based indirect immune-fluorescence assay (Fig. 1). HEK293 cells were co-transfected with full-length human GFAP and pcDNA3.1-enhanced green fluorescent protein. The cells were fixed with 4% paraformaldehyde for 20 min, rinsed with phosphate-buffered saline (PBS), and permeabilized with 0.1% Triton X-100 in PBS for 20 min to enable antibody detection after 36 h of transfection in 96-well plates. 10% PBS goat serum was used to dilute the serum/CSF by 1:10. Cells were then cultivated for 2 h at room temperature. Following three rounds of washing with 0.1% PBS Tween 20, the cells were cultivated with goat anti-human IgG (1:500; Thermo Fisher Scientific, Waltham, MA, USA) for 30 min before being cleaned once more with 0.1% PBS Tween 20 and examined using immunofluorescence microscopy. Each was graded independently by two masked evaluators. The degree of immunofluorescence in each sample was compared directly to the immunofluorescence of control samples and non-transfected cells before two impartial assessors, wearing masks, assessed each sample as positive or negative. Following confirmation, the titers of the positive specimens were determined by serially diluting them three times, from 1:100 to 1:1000. The sample dilution value for which specific fluorescence was just scarcely discernible but certainly recognizable was defined as the final titer and expressed as the corresponding dilution value. A titer of 1:10 or above was considered a positive result.

### Standard protocol approvals, registrations, and patient consents

The study was approved by the Institute Review Board of Beijing Tongren Hospital, Capital Medical University, and all subjects provided written informed consent. GFAP-Ab testing was performed as part of the clinical routine evaluation; thus, no other specific consent was required. All methods were carried out in accordance with relevant guidelines and regulations.

## Results

### Clinical profile

Four patients (9.3%) detected GFAP-Ab positive in serum and/or CSF (serum positive, CSF unavailable, 2; serum positive, CSF negative, 1; both serum and CSF positive, 1) after study inclusion (Patient 1 at day 10 of the first attack, Patient 2 at 3 months of the second attack, Patient 3 at 2 months of the first attack, Patient 4 at 1 month of the second attack) (Table 1). All four patients presented with unilateral visual loss during each onset without other neurological events. Three GFAP-Ab-positive patients (1, 2, and 4) experienced severe visual loss (BCVA ≤ 0.1), while Patient 3 had a mild visual loss (BCVA, 0.8). Two patients (1 and 3) had their first attack and two (2 and 4) manifested recurrent ON (second attack) at the time of sampling (Table 1).

**Table 1:** Demographic, clinical features, and MRI findings of patients with GFAP-Ab-positive optic neuritis

References	Patient 1	Patient 2	Patient 3	Patient 4	
Age	37	57	25	66	
Sex	F	F	M	F	
Systemic Symptoms	BS	No	No	No	
Number of ON Episodes before SIS	1	2	1	2	
Duration of the Last ON Episode Before SIS	7 d	3 m	2 m	1 m	
Treatment Before SIS	No IT	C	No IT	No IT	
Treatment After SIS	No IT	C, AZA	No IT	No IT	
GFAP-Ab (Serum/CSF) at the SIT	Serum, 1:32 positive; CSF, unavailable	Serum, 1:32 positive; CSF, unavailable	Serum, 1:32 positive; CSF, negative	Both, 1:32 positive	
BCVA at the Nadir of the Last ON Episode	L, HM (DE) R, 1.2	L, NLP R, NLP (DE)	L, 0.8 (DE) R, 1.0	L, 0.1 (DE) R, 1.0	
VF of the Last ON Episode	Constriction	Unable to check	Diffuse defect	Diffuse defect	
Optic Disc at the SIT	Bilateral mild edema	Pale	Pale	Pale	
Optic Nerve MRI at the SIT	Enlargement, hyperintensity, and significant enhancement in the light orbital segment	Hyperintensity and enhancement in the right intraorbital segment and bilateral extensive perioptic nerve	Hyperintensity in the left intraorbital segment and mild enhancement in the intraorbital and intracranial segment	Hyperintensity and atrophy in the left intraorbital, intracanal and intracranial segment	
Brain MRI at the SIT	N	Extensive meningeal enhancement	N	N	
OCT at the SIT	Bilateral pRNFLthickened, Normal GCIPL	Global pRNFL and GCIPL thinning	Global pRNFL and GCIPL thinning	Global pRNFL and GCIPL thinning	
FFA at the SIT	N	N	N	N	
Associated Positive Autoimmune Ab	Thyroglobulin Ab	Anti-SSA Ab, anti-Jo-1 Ab	N	N	
CSF	N	N	N	N	

Abbreviations: GFAP = glial fibrillary acidic protein; Ab = antibody; F = female; M = male; BS = Behcet’s syndrome; ON = optic neuritis; SIS = study inclusion; SIT = study inception; d = day; m = month; DE = Diseased eye; L = left eye; R = right eye; BCVA = Best Corrected Visual Acuity; VF = Visual Field; HM = hand movement; NLP = no light perception; MRI = magnetic resonance imaging; N = normal; OCT = optical coherence tomography; pRNFL = peripapillary retinal nerve fiber layer; GCIPL = ganglion cell-inner plexiform layer; FFA = fundus fluorescein angiography; FL = fluorescent leakage; SSA = serum anti-Sjögren’s-syndrome-related antigen A; CSF = cerebrospinal fluid; IT = immunotherapy; C = corticosteroid; AZA = azathioprine

### Coexisting systemic disorders and/or tumor

Patient 1 diagnosed with Behcet’s syndrome had a history of recurrent oral ulcers and acne-like rashes for more than 3 years. No systemic disorders were found in the other three patients, and no tumor was found in any of the patients.

### MRI findings

MRI showed optic nerve hyperintensity on T2 FLAIR images in all GFAP-Ab-positive patients, and orbital section involvement was the most common. Three patients (1, 2, and 3) had corresponding optic nerve enhancement. In addition, Patient 2 had bilateral extensive perioptic and meningeal enhancement but without linear perivascular radial enhancement. MRI showed no optic chiasma involvement in all four patients (Table 1).

### Serology results

Patient 1 had a raised thyroglobulin Ab (59.2 IU/mL) level. Patient 2 had positive serum anti-Sjögren’s-syndrome-related antigen A (SSA) and anti-Jo-1 Ab. Other laboratory test results were normal or negative in all GFAP-Ab-positive patients (Table 1).

### Treatment and visual outcomes

Patient 2 had received a high-dose intravenous corticosteroid treatment followed by a prolonged oral course at another hospital and was given azathioprine when she came to our hospital 3 months after her second episode. The other three patients did not receive immunotherapy, because the visual acuity of Patient 1 recovered spontaneously and Patients 3 and Patients 4 were not in the acute phase at presentation.

The BCVA of Patient 1 returned to 1.2 spontaneously two weeks after symptom onset. The BCVA of Patient 2 returned to 0.5, while that of Patient 3 and Patient 4 did not recover four months later (Table 2).

**Table 2:** Short-term Outcomes of patients with GFAP-Ab-positive optic neuritis

References	Patient 1	Patient 2	Patient 3	Patient 4
Follow-up Time	6 m	4 m	4 m	4 m
Recurrence During Follow-up	Yes, 7weeks	No	No	No
Follow-up Serum GFAP-Ab	Negative (6 m)	Negative (4 m)	1:32 positive (1 m)	No
Follow-up BCVA	L, 1.2 R, 1.2	L, NLP R, 0.5	L, 0.8 R, 1.0	L, 0.2 R,1.0
Follow-up VF	Normal	Unable to check	Diffuse defect	Diffuse defect
Follow-up Optic Nerve MRI	No	Enhancement in the right intracranial segment and bilateral extensive perioptic nerve	No	No
Follow-up OCT	N	Global pRNFL and GCIPL thinning	Global pRNFL and GCIPL thinning	Global pRNFL and GCIPL thinning

Abbreviations: GFAP = glial fibrillary acidic protein; Ab = antibody; m = month; L = left eye; R = right eye; BCVA = Best Corrected Visual Acuity; VF = Visual Field; NLP = no light perception; MRI = magnetic resonance imaging; N = normal; OCT = optical coherence tomography; pRNFL = peripapillary retinal nerve fiber layer; GCIPL = ganglion cell-inner plexiform layer

### Clinical and OCT follow-up

**Fig. 2:**
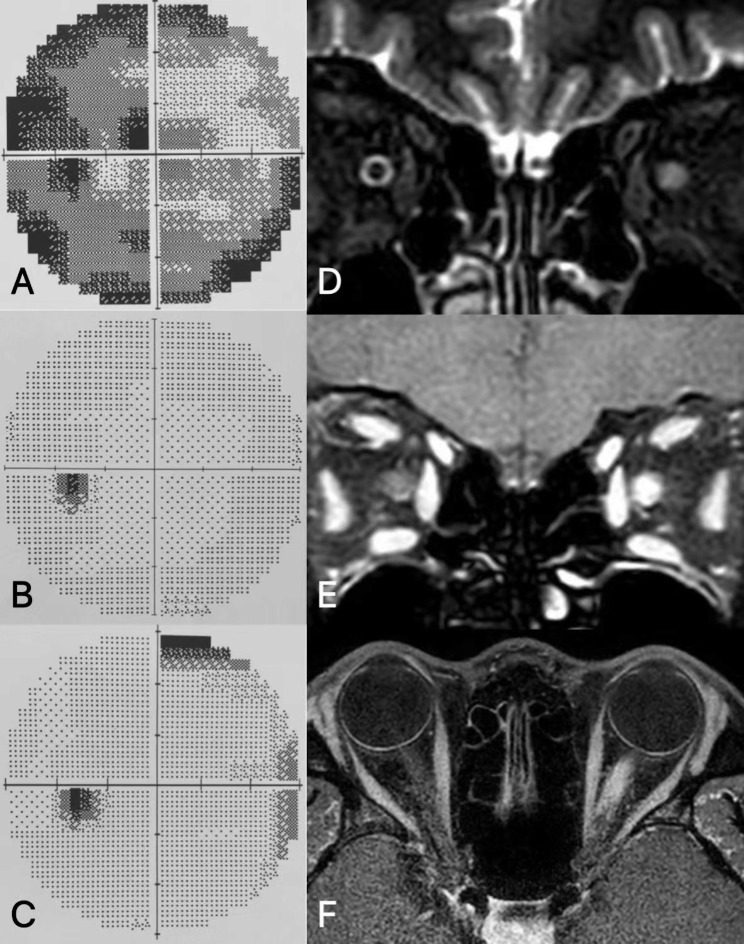
Evolution of visual field and optic nerve MRI in Patient 1. Automated visual field tests showed diffuse defects on day 7 after onset (**A**) that then resolved to normal spontaneously 2 weeks later (**B**). Seven weeks later, there were peripheral visual field defects when disease relapse occurred (**C**). MRI was performed on day 13 following onset, and the patient was not treated with corticosteroids before the scan. MRI revealed left optic nerve enlargement and hyperintensity in the orbital segment on coronal short tau inversion recovery image (**D**) and enhancement on postcontrast coronal (**E**) and axial T1 (**F**).

During follow-up (mean 4.5 ± 1 months), only Patient 1 developed a new ON episode (GFAP-Ab not tested) seven weeks after the first onset, which resolved spontaneously within a few days (Fig. 2). No patient developed other neurological events or systemic symptoms (Table 2).

OCT showed normal peripapillary retinal nerve fiber layer (pRNFL) and ganglion cell-inner plexiform layer (GCIPL) thickness in patient 1 six months later (Table 2).

## Discussion

The present study found that GFAP-Ab-positive ON can be isolated, unilateral, and relapsing in nature, contrasting the results of previous studies [6, 9–11]. One previous study described 10 GFAP-Ab-positive patients with bilateral optic disc edema who were typically visually asymptomatic [6]. In a Chinese study, 12 of 19 CSF GFAP-Ab-positive patients demonstrated abnormal vision, and severe vision loss occurred in only one case [9]. Importantly, previous research did not use optic nerve MRI or provide clinical data. White et al. reported a GFAP-Ab-positive patient who was blind in both eyes with bilateral disc swelling [10]. Her MRI revealed diffuse bilateral enlargement and enhancement of the optic nerves, which appeared 8 weeks after onset; however, her initial MRI was normal. Michael et al. reported a GFAP-Ab-positive patient who demonstrated unilateral vision loss, and the MRI demonstrated diffuse bilateral optic nerve and chiasm enhancement [11]. Importantly, all patients in previous studies demonstrated concomitant encephalitis or myelitis. The distinctive clinical characteristics of our GFAP-Ab-positive patients suggest alternative pathogeneses.

This study found that the positive GFAP-Ab was more common in serum than in CSF. Patient 3 tested positive for serum GFAP-Ab twice, despite a negative CSF test. In contrast to this, previous studies had shown that sensitivity and specificity for meningoencephalomyelitis are greater for GFAP-Ab CSF testing [2, 8]. Mayo Clinic reported 102 GFAP-Ab-positive patients, among 49 patients with both serum and CSF testing, 92% were GFAP-Ab positive in CSF, but just 45% were positive in serum. In their study, the frequency of meningoencephalomyelitis was more common among those with CSF positivity (94%) than among those with serum positivity only (61%) [2]. Another Mayo Clinic study [6] showed that among 10 patients with both bilateral optic disc edema and GFAP-Ab-positive meningoencephalitis, 100% (7/7) were GFAP-Ab positive in CSF, and 8 of 9 (89%) were positive in serum. We speculated that the detective rate of GFAP-Ab may increase in serum when the optic nerve is damaged. Since the sensitivity and specificity of GFAP-Ab in serum and CSF of isolated ON patients are unknown, we suggest that both serum and CSF GFAP-Ab should be tested in ON patients.

Patient 1 met the International Criteria for Behcet’s Disease (ICBD) and was diagnosed with Behcet’s ON [12, 13]. Behcet’s disease may present with isolated attacks and frequently with severe visual loss [14–16]. It has been reported that most patients require steroids and immune suppression, however, two patients required no initial treatment, and their conditions spontaneously resolved in previous studies [14], mirroring that of Patient 1. pRNFL and GCIPL in Patient 1 had not thinned 6 months after onset, differing from the typical features of demyelinating disease. Behcet’s syndrome can also be characterized by other autoantigens such as those of endothelial cells, enolase, and retinal S-antigen [17]. It is unclear if GFAP is another autoantigen in Behcet’s syndrome; therefore, further studies are warranted.

In our present study, thyroglobulin Ab was raised in Patient 1, and serum anti-SSA and anti-Jo-1 Ab were positive in Patient 2. Comorbid autoimmune diseases are apparent in approximately 20% of patients with GFAP-A, including autoimmune thyroid disease and rheumatoid arthritis [2]. Of the 16 GFAP-Ab-positive patients, one patient presented thyroglobulin Ab, and two patients presented thyroperoxidation-specific IgG, as reported previously [1]. Nonneural antibodies (e.g., ANA, SSA, and dsDNA Abs) were encountered in 75% of a Chinese cohort study [9]. No patients with anti-Jo-1 Ab have been reported in previous studies. Reassessment should be done to identify autoantibody-related disease entities.

The current study has some limitations. 11.6% of 43 patients did not test for GFAP-Ab in CSF, which may demonstrate a lower positive detection rate for GFAP-Ab. The application of corticosteroids before sampling and detection in the non-acute phase of ON episodes in some patients may also lead to a lower antibody positivity rate. Finally, the retrospective nature and small sample size of the present study were insufficient to describe the overall pattern of GFAP-Ab-positive ON.

In conclusion, GFAP-Ab is rare in patients with ON and may manifest as isolated, relapsing ON. This suggests that the GFAP-A spectrum may be broader and include isolated ON. Comorbid autoimmune diseases or autoantibodies are common in GFAP-A, Behcet’s syndrome may be one of the potential etiologic clues.

## Data Availability

All data generated and analyzed during this study are included in this article.

## Abbreviations

Ab: Antibody
ANA: Antinuclear Ab
AQP4: Aquaporin-4
BCVA: Best corrected visual acuity
CSF: Cerebrospinal fluid
dsDNA: Anti-double-stranded DNA
FFA: Fundus fluorescein angiography
GCIPL: Ganglion cell-inner plexiform layer
GFAP (-A / -Ab): Glial fibrillary acidic protein (-astrocytopathy / -antibody)
ICBD: International Criteria for Behcet’s Disease
IgG: Immunoglobin G
MOG: Myelin oligodendrocyte glycoprotein
MRI: Magnetic resonance imaging
OCT: Optical coherence tomography
ON: Optic neuritis
PBS: Phosphate-buffered saline
pRNFL: Peripapillary retinal nerve fiber layer
SSA: Sjögren’s-syndrome-related antigen A
VF: Visual field

## References

[1] Fang B, McKeon A, Hinson SR, Kryzer TJ, Pittock SJ, Aksamit AJ (2016). Autoimmune glial fibrillary acidic protein astrocytopathy: a novel meningoencephalomyelitis. JAMA Neurol.

[2] Flanagan EP, Hinson SR, Lennon VA, Fang B, Aksamit AJ, Morris PP (2017). Glial fibrillary acidic protein immunoglobulin G as biomarker of autoimmune astrocytopathy: analysis of 102 patients. Ann Neurol.

[3] Kimura A, Takekoshi A, Yoshikura N, Hayashi Y, Shimohata T (2019). Clinical characteristics of autoimmune GFAP astrocytopathy. J Neuroimmunol.

[4] Kunchok A, Zekeridou A, McKeon A (2019). Autoimmune glial fibrillary acidic protein astrocytopathy. Curr Opin Neurol.

[5] Gao X, Tang Y, Yang GD, Wei W (2021). Autoimmune glial fibrillary acidic protein astrocytopathy Associated with Area Postrema Syndrome: a Case Report. Front Neurol.

[6] Chen JJ, Aksamit AJ, McKeon A, Pittock SJ, Weinshenker BG, Leavitt JA (2018). Optic Disc Edema in Glial Fibrillary Acidic protein autoantibody–positive meningoencephalitis. J Neuroophthalmol.

[7] Petzold A, Fraser CL, Abegg M, Alroughani R, Alshowaeir D, Alvarenga R (2022). Diagnosis and classification of optic neuritis. LANCET NEUROL.

[8] Bennett JL, Costello F, Chen JJ, Petzold A, Biousse V, Newman NJ, Galetta SL. Optic neuritis and autoimmune optic neuropathies: advances in diagnosis and treatment. Lancet Neurol. 2022-09–22. 10.1016/S1474-4422(22)00187-9 36155661

[9] Long Y, Liang J, Xu H, Huang Q, Yang J, Gao C (2018). Autoimmune glial fibrillary acidic protein astrocytopathy in chinese patients: a retrospective study. Eur J Neurol.

[10] White D, Mollan SP, Ramalingam S, Nagaraju S, Hayton T, Jacob S (2019). Enlarged and enhancing Optic Nerves in Advanced Glial Fibrillary Acidic protein meningoencephalomyelitis. J Neuroophthalmol.

[11] Han MM, Sheils CR, Crow RW (2022). Vision loss Associated with Autoimmune glial fibrillary acidic protein astrocytopathy. J Neuroophthalmol.

[12] International Team for the Revision of the International Criteria for Behçet’s disease (ITR- ICBD) (2014). The International Criteria for Behçet’s disease (ICBD): a collaborative study of 27 countries on the sensitivity and specificity of the new criteria. J Eur Acad Dermatol Venereol.

[13] International Study Group for Behçet’s disease (1990). Criteria for diagnosis of Behçet’s disease. International Study Group for Behçet’s disease. Lancet.

[14] Kidd DP (2013). Optic neuropathy in Behçet’s syndrome. J Neurol.

[15] Akdal G, Toydemir HE, Saatci AO (2018). Characteristics of optic neuropathy in Behçet disease. Neurol Neuroimmunol Neuroinfamm.

[16] Yang Q, Sun L, Wang Q (2019). Primary optic neuropathy in Behçet’s syndrome. MULT SCLER J.

[17] Mattioli I, Bettiol A, Saruhan-Direskeneli G, Emmi G (2021). Pathogenesis of Behçet’s syndrome: genetic, environmental and immunological factors. Front Med (Lausanne).

